# Anti-diabetic Effects of *Clostridium butyricum* CGMCC0313.1 through Promoting the Growth of Gut Butyrate-producing Bacteria in Type 2 Diabetic Mice

**DOI:** 10.1038/s41598-017-07335-0

**Published:** 2017-08-01

**Authors:** Lingling Jia, Dongyao Li, Ninghan Feng, Muhammad Shamoon, Zhenghua Sun, Lei Ding, Hao Zhang, Wei Chen, Jia Sun, Yong Q Chen

**Affiliations:** 1 State Key Laboratory of Food Science and Technology, School of Food Science and Technology, Jiangnan University, Wuxi, 214122 P. R. China; 2 Wuxi School of Medicine, Jiangnan University, Wuxi, 214122 Jiangsu P. R. China; 3 Wuxi No. 2 Hospital, Jiangsu, P. R. China; 4 Department of Biology and Chemistry, University Bremen. Leobener Str., NW 2, 28359 Bremen, Germany; 5 Department of Cancer Biology, Wake Forest School of Medicine, Winston-Salem, NC 27157 USA

## Abstract

Patients with type 2 diabetes (T2D) have decreased butyrate-producing bacteria. We hypothesized that supplementation with butyrate-producing bacteria may exert beneficial effects on T2D. The current study investigated the effects of well-characterized butyrate-producing bacteria *Clostridium butyricum* CGMCC0313.1 (CB0313.1) on hyperglycemia and associated metabolic dysfunction in two diabetic mouse models. CB0313.1 was administered daily by oral gavage to leptin^*db/db*^ mice for 5 weeks starting from 3 weeks of age, and to HF diabetic mice induced by high fat diet (HFD) plus streptozotocin (STZ) in C57BL/6J mice for 13 weeks starting from 4 weeks of age. CB0313.1 improved diabetic markers (fasting glucose, glucose tolerance, insulin tolerance, GLP-1 and insulin secretion), and decreased blood lipids and inflammatory tone. Furthermore, CB0313.1 reversed hypohepatias and reduced glucose output. We also found that CB0313.1 modulated gut microbiota composition, characterized by a decreased ratio of Firmicutes to Bacteroidetes, reduced *Allobaculum* bacteria that were abundant in HF diabetic mice and increased butyrate-producing bacteria. Changes in gut microbiota following CB0313.1 treatment were associated with enhanced peroxisome proliferator–activated receptor-γ (PPARγ), insulin signaling molecules and mitochondrial function markers. Together, our study suggests that CB0313.1 may act as a beneficial probiotic for the prevention and treatment of hyperglycemia and associated metabolic dysfunction.

## Introduction

Emerging evidence indicates that gut microbiota-host interactions play a key role in the pathophysiology of type 2 diabetes and modulates energy homeostasis, glucose homeostasis and insulin resistance^1–9^. Patients with T2D are characterized by a moderate degree of gut microbial dysbiosis, particularly manifested as a lower abundance of universal butyrate-producing bacteria and an increase in opportunistic pathogens^10^. Therefore, modulating the gut microbiota via dietary interventions, especially increasing the abundance of butyrate-producing bacteria, may offer a feasible strategy to counteract T2D and associated metabolic abnormalities^11^. However, no specific butyrate-producing bacterial strain has been investigated in the treatment of T2D.

Butyrate-producing bacteria are probiotics that preferentially ferment dietary fibers into butyrate and other short chain fatty acids (SCFAs) in the colon^11, 12^. SCFAs, particularly butyrate, could trigger the secretion of incretin hormone glucagon-like peptide 1 (GLP-1), and the beneficial effects are thought to be elicited via SCFA receptors Ffar2 and Ffar3^13–16^. It has been earlier reported that butyrate improves insulin resistance^17^ and fasting hyperglycemia^18^ by inhibiting adipocyte inflammation. However, butyrate is not widely prescribed as a therapy, primarily because of its instability and deleterious effects^19^; therefore, there is a need for newer, stable and safe butyrate products.

Among butyrate-producing bacteria, CB0313.1 is an effective agent known for its effects on maintaining intestinal mucosa, mitigating gut-associated ailments like ulcerative colitis and Crohn’s disease in clinical practice^20^. However, the potential effects of CB0313.1 in diabetes and associated metabolic dysfunction are unknown.

To this end, the current study aims to (i) elucidate the effects of CB0313.1 on diabetes and associated metabolic disorders in diabetic mice; and (ii) investigate the influence of CB0313.1 on SCFA production and the taxonomic profile of the gut microbiota. This study will help understand the role of CB0313.1 in diabetes and promote the development of probiotic-based therapies of diabetes and associated metabolic dysbiosis.

## Results

### CB0313.1 improves hyperglycemia and insulin resistance in lep^*db/db*^ Mice

**Figure 1:**
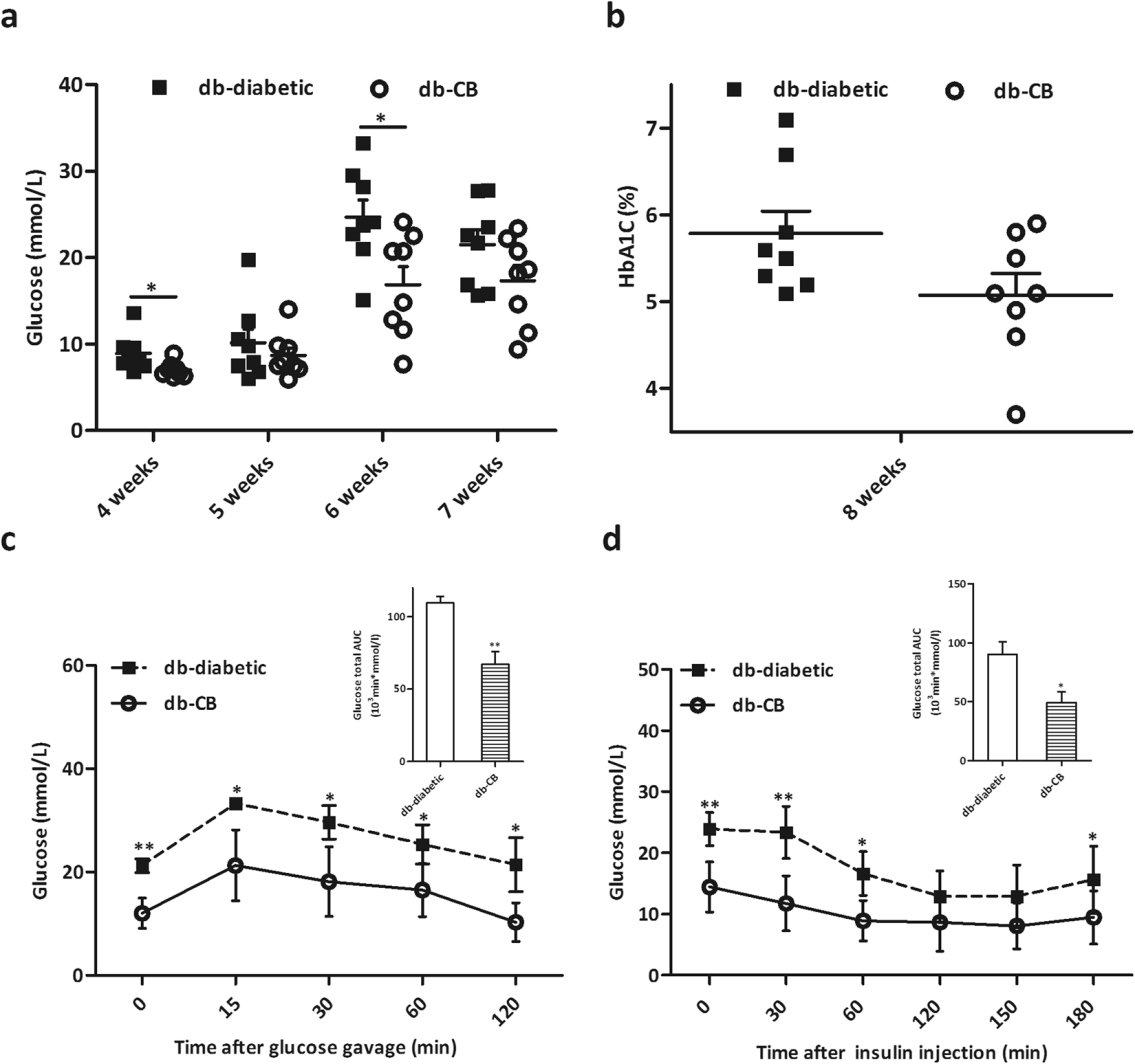
CB0313.1 improves hyperglycemia and insulin resistance in lep^*db/db*^ mice. (**a**) Glucose was tested after 6 h fasting. (**b**) HbA1C. Glucose (**c**) and insulin (**d**) tolerance tests were performed at 8 weeks. Data are mean ± SEM (n = 4–8 mice/group). *^,^ **mean *p* < 0.05, *p* < 0.01 respectively *vs* db-diabetic control by *t*-test.

To test the effects of CB0313.1 on glucose homeostasis, we performed oral glucose (OGTT) and insulin (ITT) tolerance tests. db-CB0313.1 mice (n = 8) exhibited significantly lower fasting glucose (Fig. 1a) and lower HbA1C (Fig. 1b) compared with db-diabetic mice (n = 8). In OGTT and ITT tests, db-CB0313.1 mice exhibited improved glucose tolerance at individual time points compared with db-diabetic mice with respect to area under the blood glucose response curve (AUC) (Fig. 1c). Similarly, insulin sensitivity was significantly enhanced in the db-CB0313.1 mice (Fig. 1d).

### CB0313.1 improves glucose homeostasis and reduces serum diabetic biomarkers in HF diabetic mice

**Figure 2:**
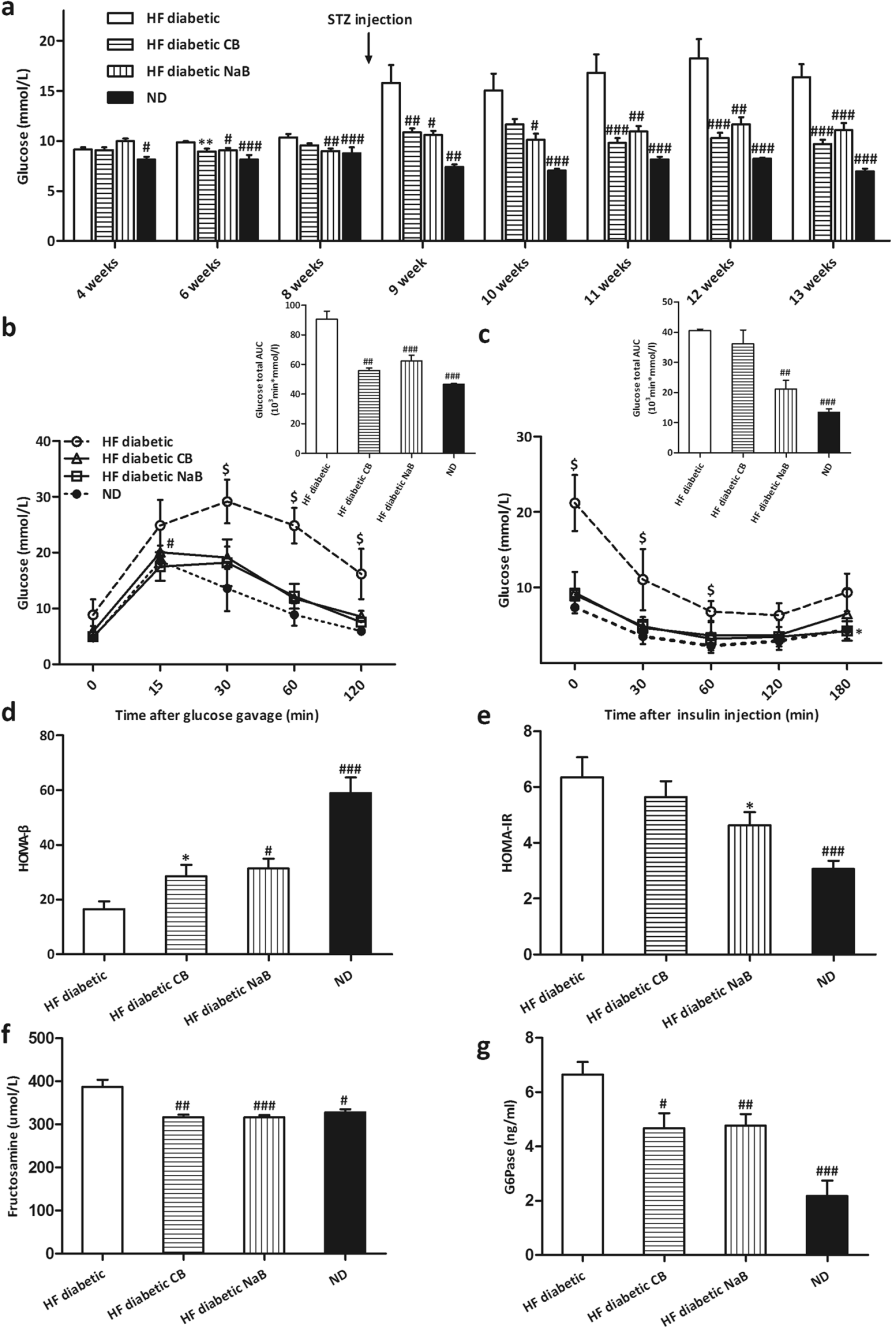
CB0313.1 improves glucose homeostasis and attenuates changes in serum diabetic biomarkers in HF diabetic mice. (**a**) Glucose was tested after 6 h fasting. Glucose (**b**) and insulin (**c**) tolerance tests with total glucose area under the curve (AUC) were performed 4 weeks after STZ injection. (**d**) Beta cell sensitivity: HOMA-β = 20 × Fasting insulin (mIU/L)/[Fasting blood glucose (mmol/L)−3.5]. (**e**) Insulin resistance: HOMA-IR = fasting blood glucose (mmol/L) × fasting insulin (mIU/L)/22.5. (**f**) Serum fructosamine. (**g**) Serum G6Pase. Data are mean ± SEM (n = 3–12 mice per group). ^#,^^##,^^###^ *p* < 0.05, *p* < 0.01, *p* < 0.001 *vs* HF diabetic control by one-way ANOVA followed by the indicated post hoc test. *^,^ **^,^ ***p < 0.05, *p* < 0.01, *p* < 0.001 *vs* HF diabetic control by *t*-test. ^$^ *p* < 0.05 *vs* all groups. *HF diabetic:* high fat diet and streptozotocin, *HF diabetic CB:* high fat diet and streptozotocin plus CB0313.1, *HF diabetic NaB:* high fat diet and streptozotocin plus NaB, *ND:* Normal diet.

To confirm the effects of CB0313.1 on glucose homeostasis, the HF diabetic mice were used to repeat the experiment. Butyrate (NaB) was used as a positive control. Similarly to the results obtained in the lep^*db/db*^ mice, CB0313.1-treated mice exhibited significantly lower fasting glucose (Fig. 2a) and better oral glucose tolerance at individual time points with respect to the area under the blood glucose response curve (AUC) compared to HF diabetic controls (Fig. 2b). Similarly, insulin sensitivity was also significantly enhanced in CB0313.1-treated mice (Fig. 2c). In CB0313.1-treated mice, homeostasis model assessment for beta cell sensitivity (HOMA-β) was higher (Fig. 2d *p* = 0.023), and homeostasis model assessment for insulin resistance (HOMA-IR) was lower than HF diabetic control (Fig. 2e). Furthermore, CB0313.1-treated mice exhibited significantly lower serum fructosamine (Fig. 2f *p* = 0.0005) and glucose-6-phosphatase (G6Pase) levels (Fig. 2g *p* = 0.0002). Taken together, these data suggest CB0313.1 improves hyperglycemia and insulin resistance in HF diabetic mice.

### CB0313.1 mitigates energy metabolic dysfunction via reducing inflammatory tone in adipose tissue in HF diabetic mice

**Figure 3:**
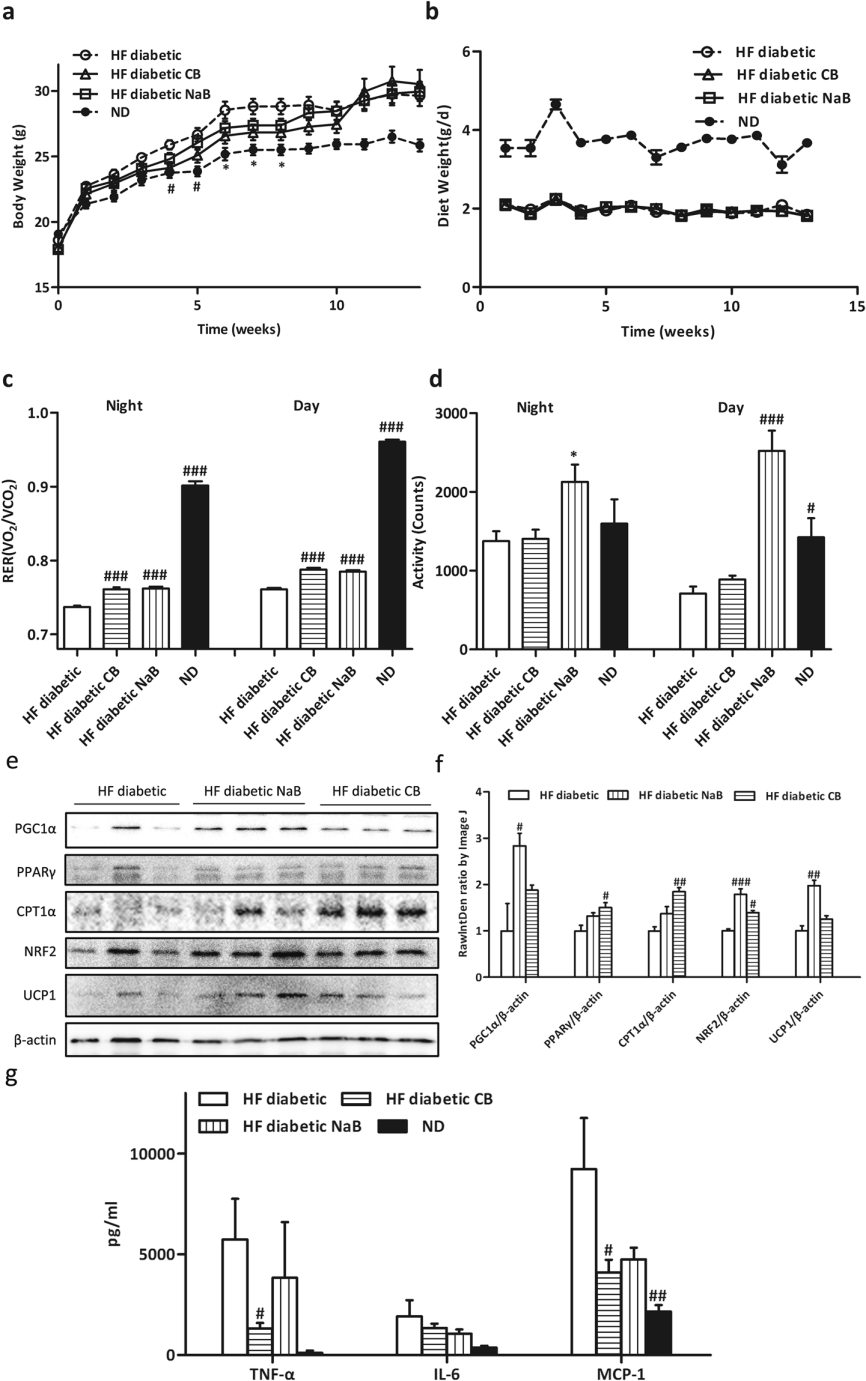
CB0313.1 mitigates energy metabolic dysfunction via reducing inflammatory tone in adipose tissue in HF diabetic mice. Energy expenditure was examined using a metabolic chamber at 17 weeks of age. Body weight (**a**) and food intake (**b**) were monitored weekly; (**c**) Substrate utilization is expressed by respiratory exchange ratio (RER), the ratio of O_2_ consumption to CO_2_ exhalation volume; (**d**) Spontaneous physical activity; (**e** and **f** ) Western blot analysis of mitochondrial metabolism proteins: PGC1α, PPARγ, CPT1α, NRF2, UCP1 and β-actin as housekeeping protein (n = 3); (**g**) Related protein levels of inflammatory markers determined by ELISA. Data are mean ± SEM (n  = 3–8 mice per group). ^#,^^##,^^###^ *p* < 0.05, *p* < 0.01, *p* < 0.001 *vs* HF diabetic control by one-way ANOVA followed by the indicated post hoc test. *^,^ **^,^ ***p < 0.05, *p* < 0.01, *p* < 0.001 *vs* HF diabetic control by *t*-test. *HF diabetic:* high fat diet and streptozotocin, *HF diabetic CB:* high fat diet and streptozotocin plus CB0313.1, *HF diabetic NaB:* high fat diet and streptozotocin plus NaB, *ND:* Normal diet.

Next we investigated the therapeutic effect of CB0313.1 on energy metabolic dysfunction in HF diabetic mice. CB0313.1 treatment attenuated body weight gain in the first ten weeks (Fig. 3a), and this effect was not associated with food intake (Fig. 3b). HF diabetic mice showed a significant lower respiratory exchange ratio (RER) compared to normal-diet (ND)-fed mice, indicating an increased fat oxidation in HF diabetic controls, while CB0313.1 can partially reverse the decrease (Fig. 3c *p* < 0.0001 for both day and night), demonstrating increased oxidation of glucose and proteins in response to CB0313.1 compared to HF diabetic controls. Interestingly, an obvious increase in diurnal and nocturnal physical activity was observed only in NaB-treated mice, while the CB0313.1-treated mice exhibited similar physical activity compared to HF diabetic mice (Fig. 3d). These findings demonstrate that CB0313.1 prevents high fat diet-induced obesity, and this effect may be associated with increased glucose, proteins oxidation and decreased fat oxidation.

Given significantly increased RER and improved insulin sensitivity in CB0313.1-mice *vs* HF diabetic controls, we speculated that CB0313.1 might affect mitochondrial metabolism of adipose tissue. In CB0313.1-treated mice, we observed an increase of mitochondrial function marker PPARγ (1.50-fold, *p* = 0.0339), carnitine palmitoyltransferase-1α (CPT1α) (1.85-fold, *p* = 0.0057), nuclear factor-like 2 (NRF2) (1.39-fold, *p* = 0.0012) in epididymal adipose tissue (Fig. 3e and f). However, no significant changes were seen in the expression of peroxisome proliferator–activated receptor coactivator 1α (PGC1α) and uncoupling protein 1 (UCP1). We also found that inflammatory marker tumor necrosis factor α (TNF-α) and monocyte chemotactic protein 1 (MCP-1) decreased by 77.17% (*p* = 0.0498), 55.61% (*p* = 0.0043) respectively in CB0313.1-treated mice (Fig. 3g). Taken together, these data suggest CB0313.1 may mitigate energy metabolic dysfunction via reducing inflammatory tone and promoting mitochondrial metabolism in adipose tissue.

### CB0313.1 alleviates pancreatic damage and enhanced insulin signaling in HF diabetic mice

**Figure 4:**
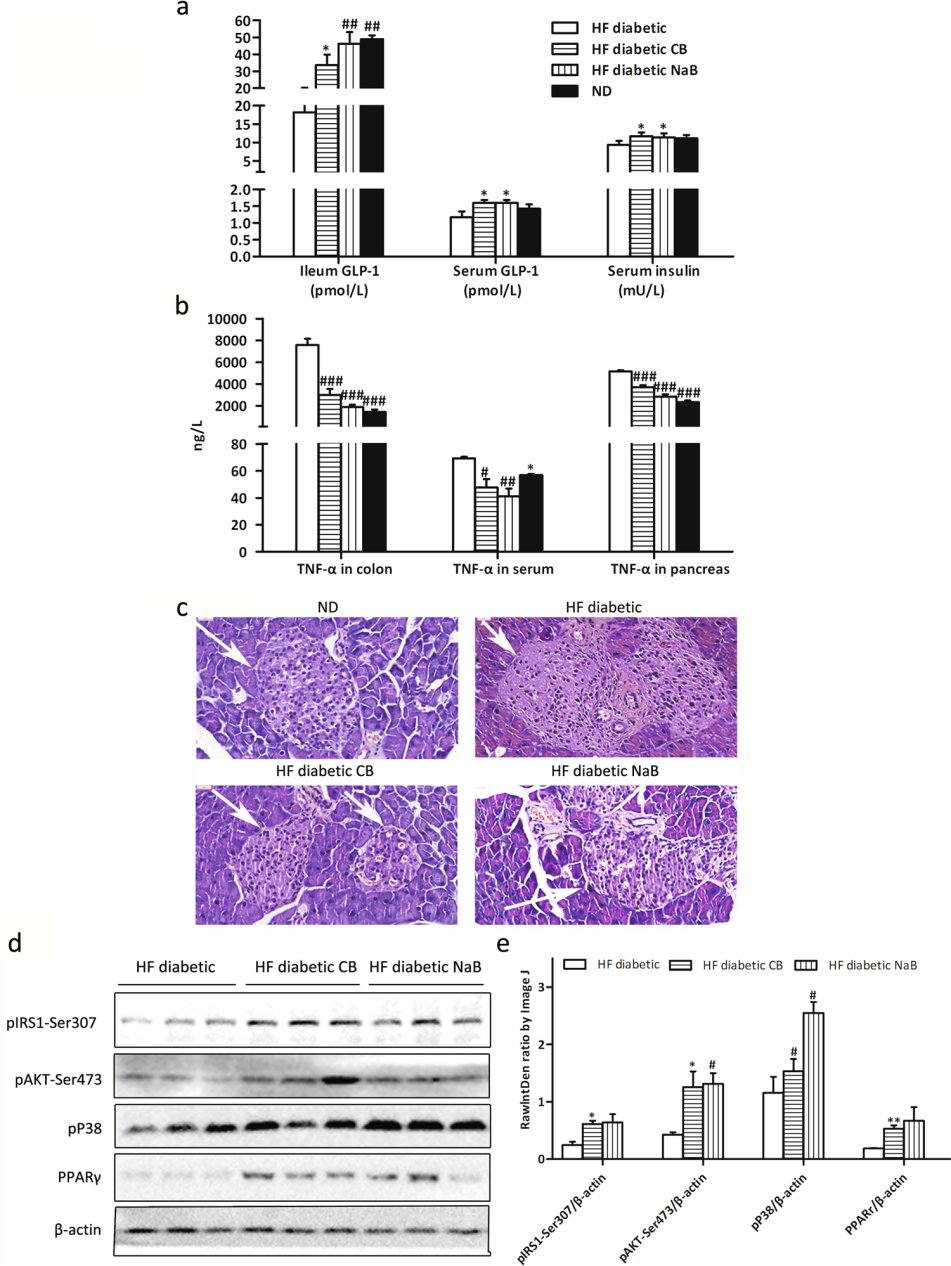
CB0313.1 alleviated pancreatic damage and enhanced insulin signaling in HF diabetic mice. (**a**) Ileum and serum GLP-1, and insulin; (**b**) Colon, serum and pancreas TNF-α; (**c**) H&E staining of pancreas was performed to observe the morphology of islets; (**d**) Insulin signaling and PPARγ in liver by Western blot; (**e**) Grey value analysis of western blot by Image J. Data are mean ± SEM (n = 3–12 mice per group). ^#,^^##,^^###^ *p* < 0.05, *p* < 0.01, *p* < 0.001 *vs* HF diabetic control by one-way ANOVA followed by the indicated post hoc test. *^,^ **^,^ ***p < 0.05, *p* < 0.01, *p* < 0.001 *vs* HF diabetic control by *t*-test. *HF diabetic:* high fat diet and streptozotocin, *HF diabetic CB:* high fat diet and streptozotocin plus CB0313.1, *HF diabetic NaB:* high fat diet and streptozotocin plus NaB, *ND:* Normal diet.

Next, we studied the effects of CB0313.1 on pancreatic beta cell destruction. CB0313.1-treated mice exhibited significantly increased GLP-1 and insulin secretion (Fig. 4a *p* = 0.0393, 0.0307 and 0.0322 respectively). In addition, significantly decreased TNF-α levels were observed in the colon, serum and pancreas, indicating that CB0313.1 attenuated systemic inflammation (Fig. 4b *p* < 0.0001, *p* = 0.0373 and *p* < 0.0001).

Moreover, the morphology of pancreas was improved in CB0313.1-treated mice. Pancreatic islet cells of ND-fed mice were regularly distributed and of uniform size, while in HF diabetic controls, the pancreatic islet cell size was inconsistent, with hyperplasia around the pancreatic duct, and pancreatic duct expansion. These conditions were markedly improved in CB0313.1-treated mice (Fig. 4c).

Subsequently, the insulin signaling pathway was investigated in the liver by examining phosphorylation of insulin receptor substrate 1 (pIRS1)-Ser307 and phosphorylation of protein kinase B (pAKT)-Ser473. Both markers of insulin signaling were increased in CB0313.1-treated mice (increased by 2.47-fold, *p* = 0.0131, and 2.93-fold, *p* = 0.039, respectively), suggesting an activated insulin signaling pathway. In addition, PPARγ was increased significantly by 2.84-fold (*p* = 0.0041), suggesting the hypoglycemic mechanism of CB0313.1 may be associated with an attenuation of energy metabolic dysfunction and obesity (Fig. 4d and e).

### CB0313.1 lowers liver damage and gluconeogenesis-related genes in HF diabetic mice

**Figure 5:**
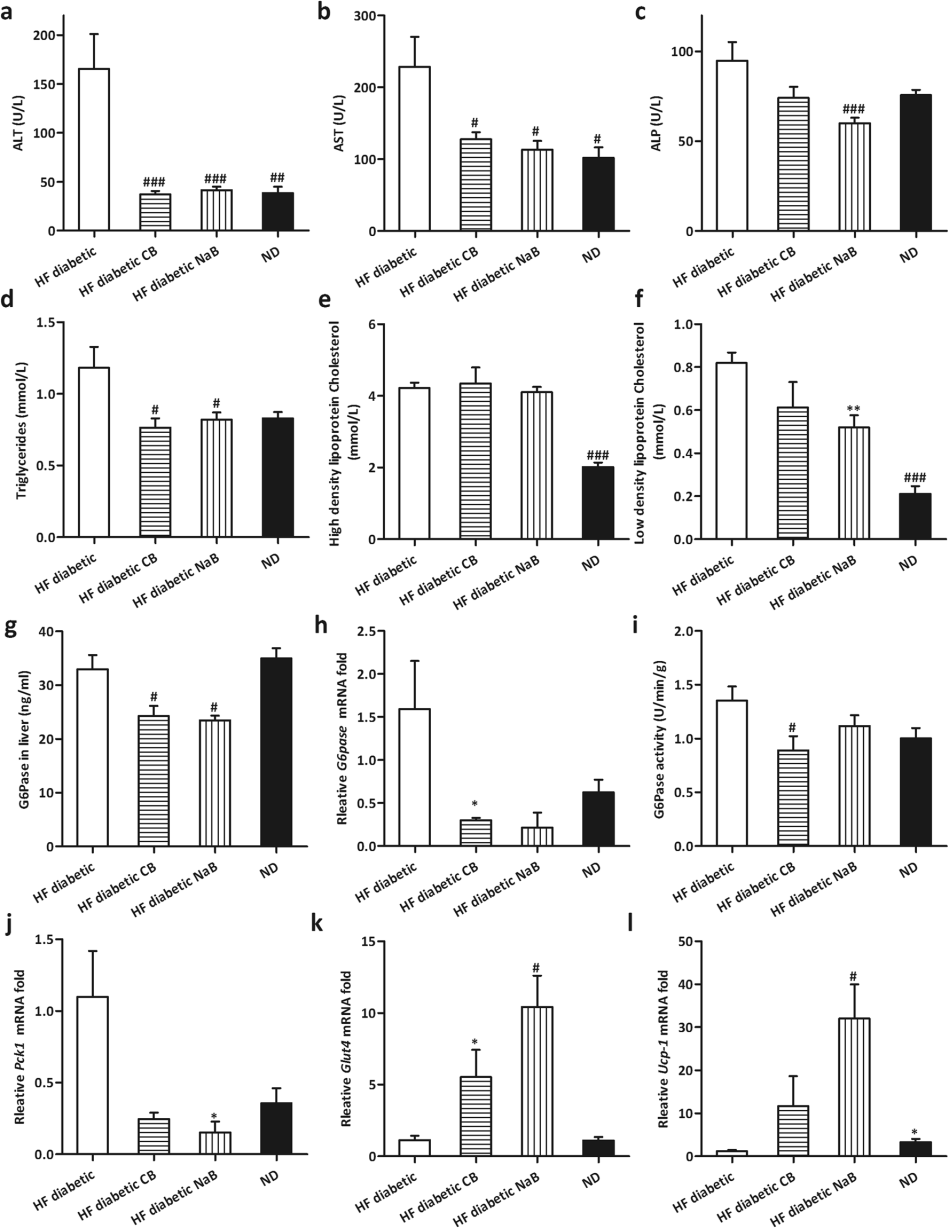
CB0313.1 Lowered Liver Damage and Gluconeogenesis-related Genes in HF Diabetic Mice. Serum ALT (**a**), AST (**b**), ALP (**c**), Triglycerides (**d**), High density lipoprotein Cholesterol (**e**) and Low density lipoprotein Cholesterol (**f**). (**g**) G6Pase protein level in liver determined by ELISA. (**h**) *G6pase* mRNA in liver. (**i**) G6Pase activity in liver. (**j**-**l**): The expression of other relevant gluconeogenesis genes was evaluated in the liver of mice fed on the indicated diets. Data are mean ± SEM (n = 3–12 mice per group). ^#,^^##,^^###^ *p* < 0.05, *p* < 0.01, *p* < 0.001 *vs* HF diabetic control by one-way ANOVA followed by the indicated post hoc test. *^,^ **^,^ ***p < 0.05, *p* < 0.01, *p* < 0.001 *vs* HF diabetic control by *t*-test. *HF diabetic:* high fat diet and streptozotocin, *HF diabetic CB:* high fat diet and streptozotocin plus CB0313.1, *HF diabetic NaB:* high fat diet and streptozotocin plus NaB, *ND:* Normal diet.

CB0313.1 intervention significantly reduced serum aminotransferase levels [alanine transaminase (ALT), aspartate aminotransferase (AST) and alkaline phosphatase (ALP)] compared to HF diabetic mice (Fig. 5a–c, *p* = 0.0003, 0.0116, 0.016, respectively). Furthermore, serum lipid markers such as total cholesterol (TC) and low density lipoprotein cholesterol (LDL-C) in CB0313.1-treated mice were also reduced *vs* HF diabetic control mice (Fig. 5d–f). Taken together, these data demonstrate that CB0313.1 can attenuate hepatic damage induced by high fat diet and STZ injection.

Next, we assessed several key enzymes which affect hepatic glucose output. As shown in Fig. 5g–i, CB0313.1-treated mice exhibited reduced hepatic G6Pase at the protein, mRNA, and enzyme activity levels compared to HF diabetic controls (*p* = 0.0005, 0.0183, 0.0322, respectively). Similarly, decreased phosphoenolpyruvate carboxykinas 1 (*Pck1)* mRNA level was observed in response to CB0313.1 (Fig. 5j, *p* = 0.0025). Notably, glucose transporter 4 (*Glut4*) mRNA levels in CB0313.1 and NaB-fed mice were increased by 5.53- and 10.44-fold, respectively (Fig. 5k, *p* = 0.0006). *Ucp-1* is a key gene capable of retarding oxidative phosphorylation and thereby hindering the production of ATP^17^. Its mRNA levels in CB0313.1 and NaB-fed mice increased by 11.67- and 32.04-fold, respectively (Fig. 5l, *p* = 0.0021). These findings demonstrate that the hypoglycemic mechanism of CB0313.1 may be associated with inhibition of hepatic glucose output via the insulin signaling pathway.

### CB0313.1 restores the diabetes-induced gut microbial dysbiosis at different taxonomic levels in HF diabetic mice

**Figure 6:**
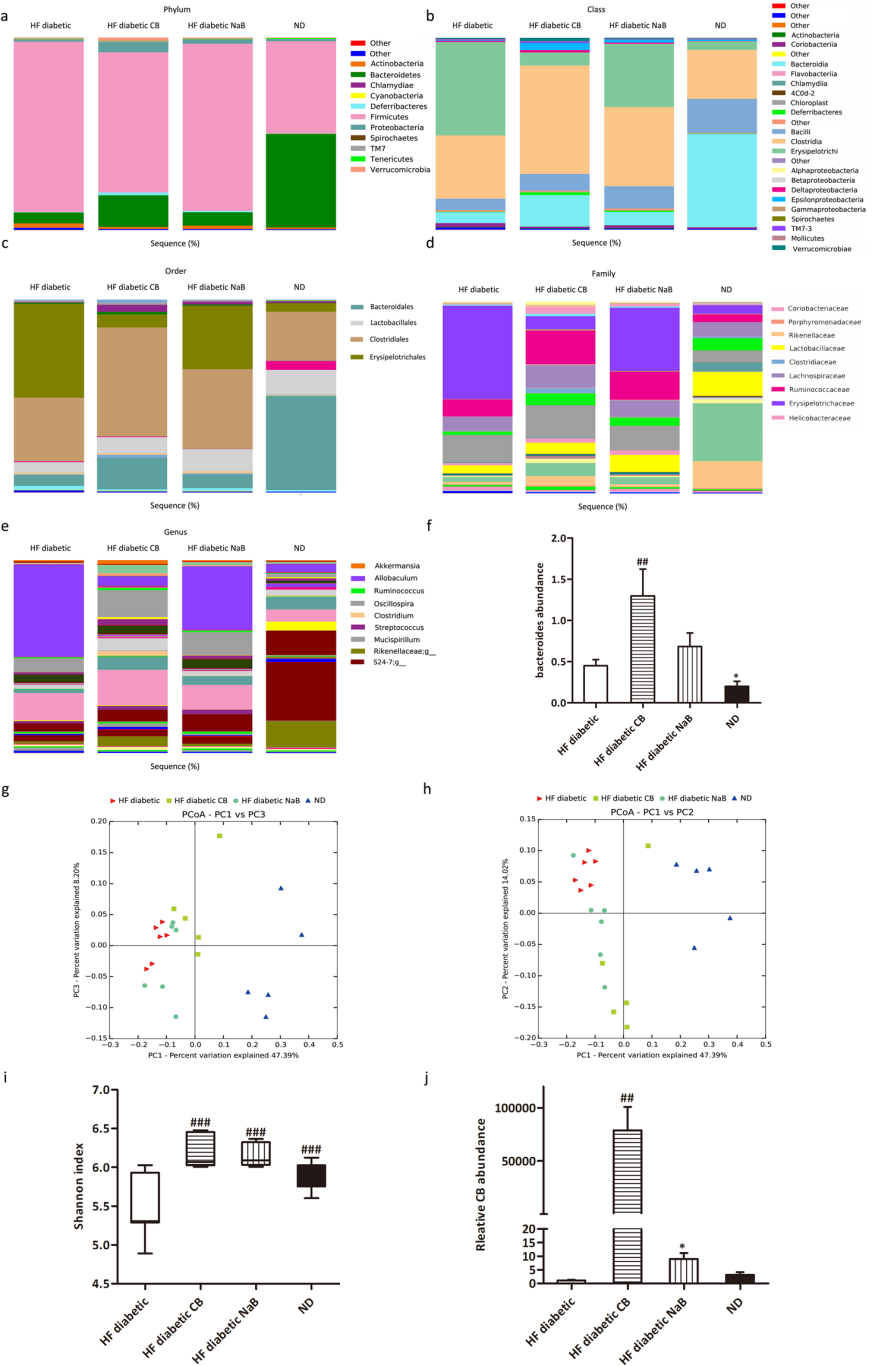
CB0313.1 restored the diabetes-induced gut microbial dysbiosis at different taxonomic levels in HF diabetic mice. (**a**) Abundance of the most important phyla in each group. Abundance of the main altered classes (**b**), orders (**c**), families (**d**), and genera (**e**) in each group. (**f**) Abundance of *bacteroides*. (**g**) and (**h**) Principal coordinate analysis (PCoA) plot of weighted UniFrac distances, each dot representing a colonic community; the percentage of variation explained by each principal coordinate is shown in parentheses. (**i**) Diversity index: Shannon index. (**j**) Relative abundance of CB0313.1. Data are mean ± SEM (n = 3–12 mice per group). ^#,^^##,^^###^ *p* < 0.05, *p* < 0.01, *p* < 0.001 *vs* HF diabetic control by one-way ANOVA followed by the indicated post hoc test. *^,^ **^,^ ***p < 0.05, *p* < 0.01, *p* < 0.001 *vs* HF diabetic control by *t*-test. *HF diabetic:* high fat diet and streptozotocin, *HF diabetic CB:* high fat diet and streptozotocin plus CB0313.1, *HF diabetic NaB:* high fat diet and streptozotocin plus NaB, *ND:* Normal diet.

16S rRNA gene sequencing data showed that CB0313.1 profoundly affected the abundance of main microbial phyla in the digestive tract. CB0313.1 was associated with a significant increase in Bacteroidetes abundance (approximately tripled, *p* < 0.0001) and a decrease in Firmicutes abundance (by 15.6%, *p* < 0.0001) compared to HF diabetic mice (Fig. 6a and Supplementary Table S3). Figure 6b and c illustrate the microbial alterations at the class and order levels, respectively. Interestingly, taxonomic groups Clostridia and Clostridiales increased consistently, indicating that CB0313.1 promoted the establishment of a protective microbiota enriched in Clostridiales (from 32.95% in HF diabetic control to 56.53% in CB0313.1-treated mice, *p* = 0.039), which were reported to resist inflammation and involved in type 2 immunity such as type 1 diabetes^21^.

At the family level, 10 out of 25 families identified were markedly changed following CB0313.1 treatment (Fig. 6d and Supplementary Table S3). Lachnospiraceae and Ruminococcaceae increased to 1.80-fold and 1.90-fold, respectively, in the CB0313.1-treated group. Major differences were observed at the level of dominant families: Clostridiaceae, Bacteroidaceae, Porphyromonadaceae, Rikenellaceae, Deferribacteraceae, Lactobacillaceae, and Helicobacteraceae increased by 12.27-fold (*p* = 0.0429), 2.17-fold (*p* = 0.0357), 0.25-fold, 1.65-fold, 6.86-fold, 0.26-fold and 7.43-fold, respectively, *vs* HF diabetic control, and Erysipelotrichaceae decreased from 42.03% in HF diabetic control to 8.15% in CB0313.1-treated mice.

Additionally, the following genera were increased in CB0313.1-treated mice: *Akkermansia* (1.33-fold), *Ruminococcus* (1.29-fold, *p* = 0.0272), *Bacteroides* (1.71-fold, *p* = 0.0357), *S24-7* (3.10-fold), *Clostridium* (from zero to 2.3%, *p* = 0.0385), *Streptococcus* (0.75-fold), *Mucispirillum* (4.96-fold), *Oscillospira* (0.80-fold, *p* < 0.0001) and *Rikenellaceae*_*-*_ *g*_*-*_ (1.63-fold) *vs* HF diabetic control (Fig. 6e,f and Supplementary Table S3). Previous data demonstrated that genera *Clostridium*, *Ruminococcus*, *Bacteroides* and *S24-7* were SCFA producers^22–24^, which were reported to exert beneficial effects on the intestinal barrier and metabolic dysfunction. Moreover, gram-negative bacteria *S24-7* and *Rikenellaceae* are involved in inflammatory pathways^25^. In addition, genera *Akkermansia* ^6^ and *Oscillospira* ^26^ have been reported to be beneficial for glucose homeostasis.

Interestingly, the genus *Allobaculum* (Firmicutes phylum; Erysipelotrichi class; Erysipelotrichales order; Erysipelotrichaceae family) was increased 10.95-fold in HF diabetic control mice compared to ND-fed mice, while CB0313.1 treatment restored it nearly to the baseline. Consequently, the genus *Allobaculum* was the strongest biomarker which was consistently detected across taxonomic levels (Fig. 6e and Supplementary Table S3, *p* < 0.0001).

Principal coordinate analysis (PCoA) showed that overall, the gut microbial community was significantly modified by CB0313.1, with all the HF diabetic mice exhibiting a dramatic shift along the same direction, while CB0313.1 could reverse the diabetes-induced variations along the first principal component (PC1) (Fig. 6g and h).

In addition, the Shannon indexes of the CB0313.1-treated group were significantly higher than for the HF diabetic control group, even higher than ND-fed mice, indicating increased diversity of the gut microbiota in the CB0313.1-treated group (Fig. 6i).

As expected, we found that supplementation with CB0313.1 effectively increased CB0313.1 in intestine by 78,823.27-fold. Strikingly, in the NaB-treated mice, the relative abundance of CB0313.1 was increased by 8.93-fold *vs* HF diabetic controls, suggesting that butyrate can promote the growth of CB0313.1 by some unknown mechanism (Fig. 6j, *p* = 0.0002).

These results suggest that CB0313.1 partially restored the diabetes-induced gut microbial dysbiosis.

### CB0313.1 upregulates butyrate production by *buk* and *butyryl-CoA* enzymes accompanied by an increase in the SCFA receptor in HF diabetic mice

**Figure 7:**
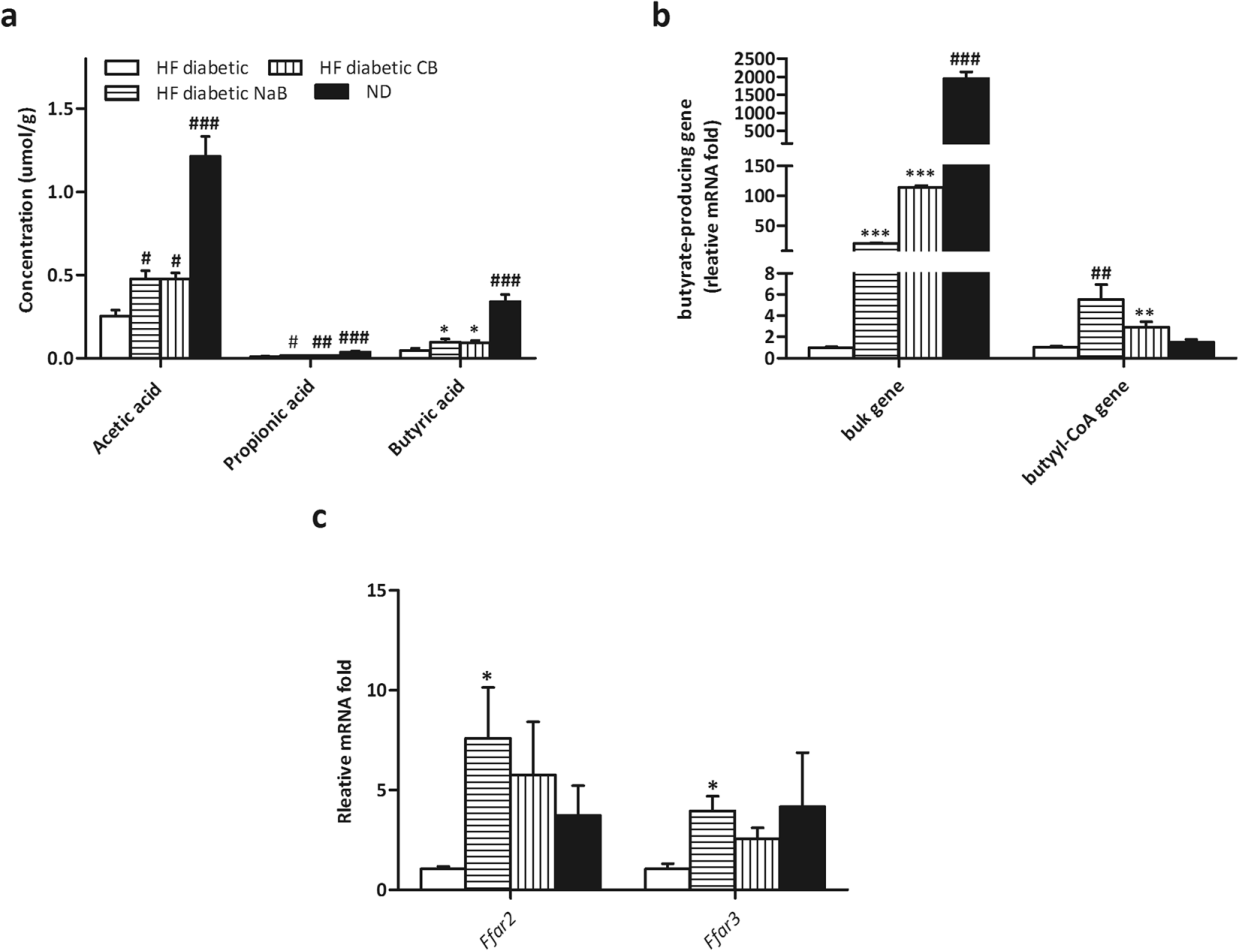
CB0313.1 upregulates butyrate production by *buk* and *butyryl*-*CoA* genes accompanied by an increase in the SCFA receptor in HF diabetic mice. (**a**) SCFA concentration in feces measured by GC-MS. Total DNA was extracted from feces and real-time PCR was performed. (**b**) Predominant butyrate producing genes: abundance of butyrate kinase (*buk*) and *butyryl-CoA* DNA in feces. (**c**) Relative mRNA expression of SCFA receptors (*Ffar2*, *Ffar3*) in colon. Data are mean ± SEM (n = 3–12 mice per group). ^#,^^##,^^###^ *p* < 0.05, *p* < 0.01, *p* < 0.001 *vs* HF diabetic control by one-way ANOVA followed by the indicated post hoc test. *^,^ **^,^ ***p < 0.05, *p* < 0.01, *p* < 0.001 *vs* HF diabetic control by *t*-test. *HF diabetic:* high fat diet and streptozotocin, *HF diabetic CB:* high fat diet and streptozotocin plus CB0313.1, *HF diabetic NaB:* high fat diet and streptozotocin plus NaB, *ND:* Normal diet.

Dietary interventions are important for butyrate synthesis in the ileum, and synthesis of SCFA, especially butyrate, is associated with multiple metabolic beneficial effects^27^. GC-MS analyses revealed that CB0313.1 restored SCFA production, which had been reduction in response to diabetes. Acetic acid, propionic acid and butyric acid were increased by 69.66%, 78.25%, and 155.55%, respectively, *vs* HF diabetic controls (Fig. 7a, *p* < 0.0001, *p* < 0.0001, *p* < 0.0001).

Typically, two genes, butyrate kinase (*buk*) and acetate CoA-transferase gene expression (*butyryl-CoA*), are used as biomarkers for the detection of butyrate-producing communities. We found that the strain CB0313.1 only carried the *buk* gene but no *butyryl-CoA* gene, suggesting that CB0313.1 produces butyrate via the *buk* pathway (data not show). However, CB0313.1 administration significantly increased not only bacteria carrying the *buk* gene (by about 20.86-fold, *p* = *0.0003*) but also those carrying the *butyryl-CoA* gene (by about 5.53-fold) (Fig. 7b, *p* = *0.0068*), demonstrating that probiotic CB0313.1 also promoted the growth of other butyrate-producing bacteria. Given the effects on SCFA production by CB0313.1, we measured the expression of SCFA receptors. The mRNA levels of SCFA receptors *Ffar2* and *Ffar3* were increased significantly in the colon of CB0313.1-treated mice (Fig. 7c, *p* = 0.0125, *p* = 0.0289).

Together, these data suggest that the probiotic CB0313.1 promotes the growth of intestinal butyrate-producing bacteria accompanied by an increase in SCFAs (especially butyrate) and their receptors.

## Discussion

This study demonstrated that CB0313.1 attenuates fasting hyperglycemia, improves insulin sensitivity by modifying the colonic microbial composition, and especially by increasing butyrate-producing bacteria, in T2D mice. To the best of our knowledge, this is the first systemic study that has analyzed the effects of CB0313.1 on the gut microbiota as well as the first study that has shown a positive effect of CB0313.1 on hyperglycemia and associated metabolic dysfunction.

Probiotics are defined as live microorganisms that confer health benefits to the host when administered in adequate amounts (FAO/WHO 2002). Despite their wide application^4, 28, 29^, probiotics have not been evaluated against diabetes, primarily due to the insufficient insight into relevant mechanisms and lack of efficacy in animal and clinical experiments^30^. T2D patients are characterized by lowered butyrate-producing bacteria^10, 31, 32^. Moreover, several effective T2D therapies, such as metformin and berberine, have been reported to be able to restore the abundance of SCFA-producing bacteria^33^. Among known butyrate-producing bacteria, CB0313.1 is a well-characterized strain of *Clostridium butyricum* ^20^.

Butyrate has been reported to improve insulin resistance for years. Because of its short half-life^19^ and potential neuronal side-effects^34, 35^, newer, more stable butyrate products with sustained/controlled drug delivery are needed for improving the pharmacokinetic and pharmacodynamic profile of butyrate, and directly or indirectly eliminate its side-effect so that ultimately it can be applied as a therapy.

We observed that CB0313.1 improved glucose homeostasis and insulin resistance (Figs 1 and 2), and we did not observe any changes in food intake between groups (Fig. 3b), suggesting that CB0313.1 modulates energy homeostasis via a mechanism other than energy intake. The dosage of NaB used in this study may have some effects on the neuron, because physical activity of NaB-treated mice was significantly increased compared to HF diabetic controls as well as ND-fed mice (Fig. 3d). Although increased physical activity might improve insulin sensitivity, due to weaken hormonal regulatory ability of diabetes patients, excess physical activity could lead to hypoglycemia^36^. On the other hand, when insulin level is low, strenuous exercise might also lead to hyperglycemia and ketoacidosis^37, 38^. Many diabetes complications, such as cardiovascular disease, diabetic nephropathy, ketoacidosis, diabetic foot, acute infection are contraindications to excess exercise^38^. While the CB0313.1-treated mice exhibited normal physical activity (Fig. 3d), suggesting that the anti-diabetic mechanism of CB0313.1 did not affect the nervous system and behavior of the mice, and that CB0313.1 represents a safer anti-diabetic agent.

Because of significantly increased RER (Fig. 3c), improved insulin sensitivity (Figs 1 and 2) and increased *Ucp1* mRNA level in liver (Fig. 5l) as well as increased levels of PPARγ, CPT1α and NRF2 in white adipose tissue (Fig. 3e and f), we speculated that CB0313.1 might affect mitochondrial metabolism.

Jing Sun *et al*. report a clostridium butyrate strain which can activate AKT in the diabetic cerebral ischemia/reperfusion (I/R) injury mouse model via gut microbiota modulation^39^. Here, we observed that CB0313.1 prevented the pancreatic damage and protected pancreatic beta cells via reducing TNFα level in pancreas (Fig. 4b) and promoting GLP-1 and insulin secretion (Fig. 4a). Insulin activated the pIRS1-pAKT-pP38-PPARγ pathway (Fig. 4d and e). GLP-1 is an insulinotropic glucoincretin hormone, known to promote β-cell survival via G protein-coupled receptor^40^ and increase beta cell proliferation through TCF7l2/Wnt pathway^41–43^. We also observed diminished levels of *G6pase* and *Pck1* (enzymes involved in gluconeogenesis) in response to CB0313.1, indicating a lower hepatic glucose output (Fig. 5). Together, these results suggest that CB0313.1 may improve host glucose homeostasis via the GLP-1/insulin/gluconeogenesis pathways.

Based on the clear effects of CB0313.1 in glucose homeostasis and insulin resistance, we continued to investigate the impact of CB0313.1 on the abundance of butyrate-producing communities, the class Clostridiales and the gut microbiota composition by high-throughput sequencing. The abundance of genera *Clostridium*, *Ruminococcus*, *Bacteroides*, and *S24-* ^22–24^, producers of SCFAs and especially butyrate, was increased in response to CB0313.1. Moreover, the Lachnospiraceae and Ruminococcaceae families (two main butyrate-producing taxonomic groups shown to be associated with healthier phenotypes)^44^ were significantly increased in CB0313.1-treated mice (Fig. 6d). Figure 5b–e illustrated the microbial alterations at the class, order, family and genus levels. Interestingly, taxonomic groups Clostridia, Clostridiales, Clostridiaceae and Clostridium were increased consistently, indicating that CB0313.1 promoted a protective microbiota, which was reported to resist inflammation in type 2 immunity^21^. Moreover, we found that CB0313.1 substantially changed the gut microbiota composition with a decreased ratio of Firmicutes to Bacteroidetes, which is negatively associated with obesity and T2D in mice^22^. At the genera level, 11 of the 31 genera identified were affected by CB0313.1 treatment. Most of them are still poorly characterized and could be further studied in the context of T2D. HF diabetic mice exhibited an obvious shift in the gut microbiome, resulting in a reduction in the abundance of genera *Rikenellaceae*, *Lactobacillus*, and *Bacteroides* and an increase in genera *Streptococcus*, *Oscillospira*, *Ruminococcus*, and *Allobaculum vs* ND-treated mice. On the other hand, CB0313.1-treated mice exhibited significant increases in genera *Bacteroides*, *Rikenellaceae*, *Streptococcus*, *Clostridium*, *Oscillospira* and decreases in genus *Allobaculum vs* HF diabetic control (Fig. 6e). The increased abundance of *Bacteroides* in CB0313.1-treated mice was in accordance with a previous study showing that berberine prevented obesity by increasing *Bacteroides* ^45^. Strikingly, only CB0313.1-treated mice exhibited an increasing trend of genus *Akkermansia* (Fig. 6e), a known beneficial bacteria in glucose tolerance^6, 46^.

Moreover, we observed that intestinal CB0313.1 increased by approximate 78,823.27-fold in CB0313.1-treated mice (Fig. 6j), confirming that CB0313.1 reached the colon effectively. Nobel *et al*. reported that *Allobaculum* was an important functional phylotype of metabolic dysbiosis^47^. In the current study, we observed an interesting phenomenon: the *Allobaculum* counts of ND-fed mice were 5.16%, in HF diabetic control the counts increased to 41.83%, while upon CB0313.1 addition, the counts decreased to 6.48%. This indicates that CB0313.1 can reverse the diabetes-induced increase in *Allobaculum* to normal level. These data demonstrated that CB0313.1 counteracted the gut microbiota dysbiosis caused by diabetes (notably *Allobaculum*, and to some extent *Bacteroides*, *Clostridium* and *Oscillospira*), and promoted the growth of anti-inflammatory bacteria in the *Clostridiales* cluster (Fig. 6a–e).

Using gene biomarkers (*buk* and *butyryl-CoA*) of butyrate-producing communities, we observed that diabetes led to a dramatic decrease in butyrate-producing bacteria, while CB0313.1 supplementation reversed the trend. Interestingly, butyrate-producing bacteria carrying the *butyryl*-CoA gene were also increased significantly by about 5.5-fold in CB0313.1-treated mice, which might be explained by a modification of the abundance of other butyrate-producing bacteria by CB0313.1 (Fig. 7). Thus, the selective modulation of gut microbial phenotypes, particularly the enrichment of butyrate-producing bacteria, may contribute to the improvement of diabetes and associated metabolic dysfunction.

As it remains to be determined whether the interaction between increased butyrate-producing bacteria and improved glucose homeostasis is direct, we have observed elevated levels of *Ffar2* and *Ffar3* SCFA receptors in the colon of CB0313.1-treated mice (Fig. 7), and increased serum and ileum GLP-1 levels (Fig. 4). We therefore speculate that CB0313.1 may exhibit beneficial effects via butyrate and its receptors *Ffar2* and *Ffar3*, which then trigger GLP-1 secretion in ileum.

**Figure 8:**
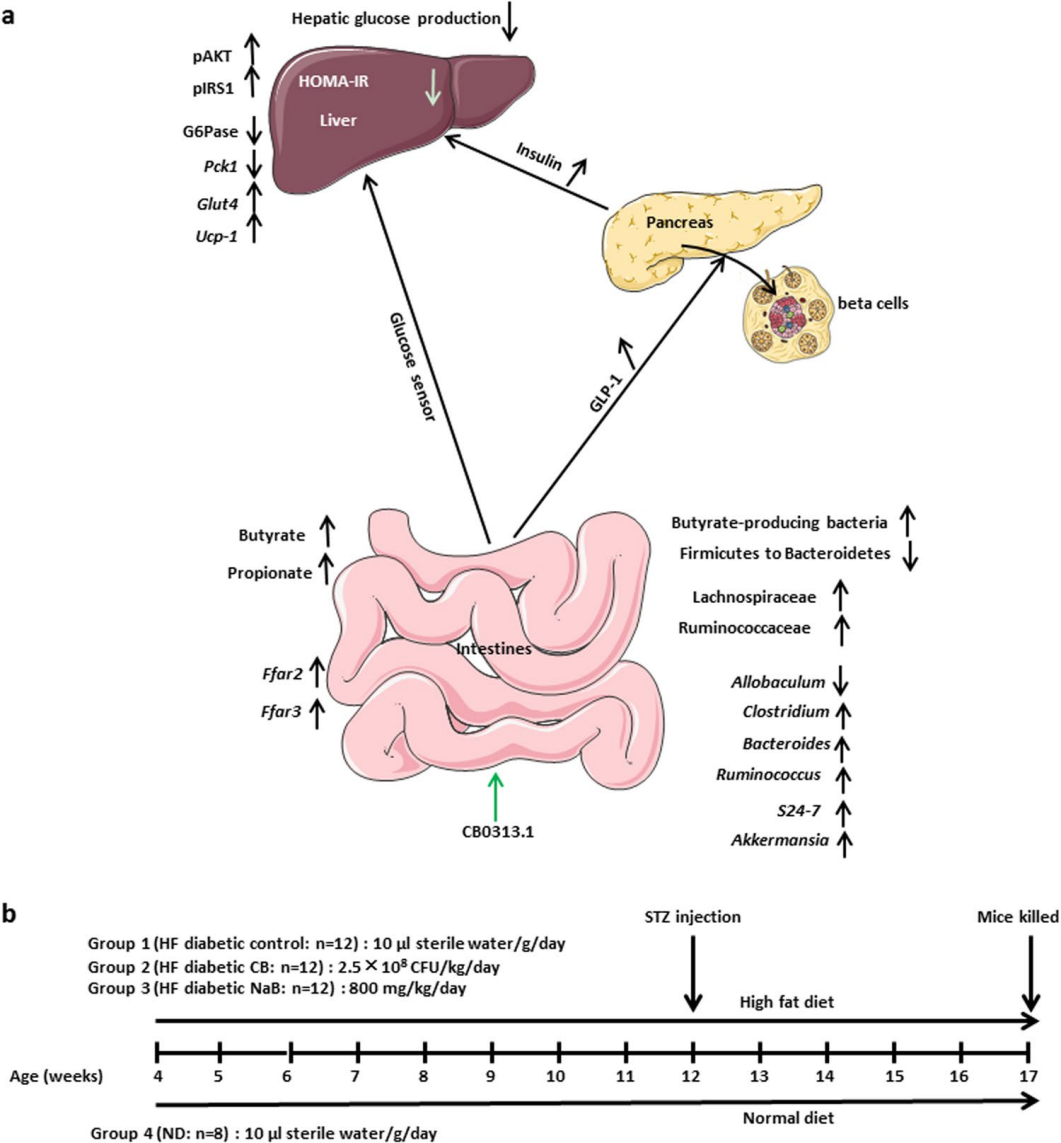
The effects of CB0313.1 on intestine, pancreas, liver and experimental design of high fat diabetic mice. (**a**) The effects of CB0313.1 on intestine, pancreas and liver. In the intestine, CB0313.1 modified the structure of the gut microbial community, characterized by a decreased ratio of Firmicutes to Bacteroidetes, reversed *Allobaculum* increase induced by diabetes and increased butyrate-producing bacteria, which led to increased SCFA production and GLP-1 secretion. In the pancreas, CB0313.1 alleviated pancreatic damage, improved the morphology of pancreatic islet, increased insulin secretion. Then, in the liver, CB0313.1 enhanced insulin signaling (pIRS1-Ser307, pAKT-Ser473) and upregulated PPARγ level, which led to reduced hepatic glucose production (G6Pase and Pck1) and insulin resistance (HOMA-IR), ultimately suppressing hyperglycemia. (**b**) Experimental design for the evaluation of the effect of CB0313.1 administration on HF diabetic mice. Male C57BL/6 J mice were randomly assigned to four groups at 4 weeks of age (n = 8–12/group): (1) HF diabetic control mice were fed HFD with sterile water by gavage daily, (2) HF diabetic CB mice were fed HFD with CB0313.1 by gavage at 2.5 × 10^8^ CFU/kg/day (suspended in sterile water), (3) HF diabetic NaB mice were fed HFD with NaB by gavage at 800 mg/kg/day (suspended in sterile water), (4) ND control mice were fed standard chow with sterile water by gavage daily. All mice of groups (1), (2), and (3) were fed HFD for 8 weeks along with the corresponding intervention and then injected with a single low dose (80 mg/kg body weight) STZ intraperitoneally. All the mice were kept on corresponding chow and intervention until euthanasia.

In conclusion, we report novel effects linking butyrate-producing bacteria CB0313.1 with alleviated hyperglycemia and delineate the underlying mechanism. CB0313.1 modulates gut microbiota composition to selectively enrich butyrate-producing bacteria, promote GLP-1 and insulin secretion, activate the GLP-1/insulin/pIRS1-pAKT-PPARγ-G6Pase/*Pck1* pathway, and reduce hepatic glucose output, which ultimately mitigates hyperglycemia in diabetic host mice. Thus, our results suggest that CB0313.1 may act as a beneficial probiotic for prevention and treatment of hyperglycemia and associated metabolic dysfunction (Fig. 8a).

## Materials and Methods

### The leptin^*db/db*^ mice and experimental design

All animal experiments were approved by the animal ethics committee of Jiangnan University, China. All animal experimental protocols were performed in accordance with the European Community guidelines (Directive 2010/63/EU). All efforts were made to minimize animal suffering.

3-week old male *db/db* mice (*BKS.Cg-Dock* ^*7m*^ + / + *Lep* ^*db*^ */Nju*; Nanjing Institute of Biological Medicine, Jiangsu, China) were randomly assigned to two groups (n = 8/group) to receive CB0313.1 (2.5 × 10^8^ CFU/kg/day, Qingdao East Sea Pharmaceutical Co. Ltd, Shandong, China) or sterile water by gavage for 5 weeks.

### The high fat diabetic mice and experimental design

Male C57BL/6 J mice (Su Pu Si Biotechnology, Co., Ltd., Suzhou, Jiangsu, China) were randomly assigned to four groups at 4 weeks of age (n = 8–12/group): (1) HF diabetic control mice were fed HFD (D12492 Research Diets, New Brunswick, NJ, in which 60% of calories are from fat) with sterile water by gavage daily, (2) HF diabetic CB mice were fed HFD with CB0313.1 by gavage at 2.5 × 10^8^ CFU/kg/day (suspended in sterile water), (3) HF diabetic NaB mice were fed HFD with sodium butyrate (NaB, Sigma 303410, St. Louis, America) by gavage at 800 mg/kg/day (suspended in sterile water), (4) Normal diet (ND) control mice were fed standard chow with sterile water by gavage daily.

All mice of groups (1), (2), and (3) were fed HFD for 8 weeks along with the corresponding intervention and then injected with a single low dose (80 mg/kg body weight) STZ (Sigma S0130, St. Louis, MO, USA) intraperitoneally^48–51^. All the mice were kept on corresponding chow and intervention until euthanasia (Fig. 8b).

### Blood glucose measurement

For leptin^*db/db*^ mice, we assayed blood glucose weekly. For HF diabetic mice induced by HFD plus STZ, we assayed blood glucose every two weeks before STZ injection, and once a week after STZ injection. Oral glucose tolerance test (OGTT) and insulin tolerance test (ITT) were performed one week before the end of the study.

### Comprehensive laboratory animal monitoring system (CLAMS) metabolic chamber

Respiratory exchange rate (RER) and spontaneous physical activity were monitored with CLAMS (Oxymax/CLAMS system, Columbus, OH, USA). The mice (16 weeks old, one week before the end of the study) were housed individually in the metabolic chamber. After 24 h of adaptation, the data in all parameters were recorded and analyzed.

### Histological evaluation

At the end of the study, harvested pancreas tissues were fixed in 4% paraformaldehyde (Sigma, HT50-1-2, St. Louis, MO, USA) overnight, washed with ddH_2_O, rehydrated with gradient ethanol solutions and embedded in paraffin. 5-μm sections were stained with Hematoxylin and Eosin (H&E) dyes following the standard procedure.

### Stool sampling, DNA extraction and sequencing

At the end of the study, stool samples were collected and immediately stored at −80 °C until the DNA extraction. Microbial genomic DNA was extracted from fecal samples using Fast DNA Spin Kit for Soil (MP Biomedicals, cat. #6560-200, California, USA) following the manufacturer’s instructions. The V4 region of 16S rRNA was PCR-amplified using primers (sense: 5′-AYTGGGYDTAAAGNG-3′; antisense: 5′-TACNVGGGTATCTAATCC-3′). Reaction conditions were 95 °C for 5 min; 95 °C for 30 s, 64 °C for 30 s, and 72 °C for 30 s, then repeat for 40 cycles; and 72 °C for 10 min. The PCR products were excised from a 1.5% agarose gel, purified by Gene Clean Turbo (MP Biomedicals, cat. #: 111102400) and quantified by Quant-iT PicoGreen dsDNA Assay Kit (Life Technologies, cat. #P7589, Carlsbad, USA) following the manufacturer’s instructions. Libraries were prepared using TruSeq DNA LT Sample Preparation Kit (Illumina, cat. #FC-121-2001, San Diego, USA) and sequenced for 500+7 cycles on Illumina MiSeq using the MiSeq Reagent Kit (500 cycles-PE, cat. #MS-102-2003).

### Real-time-PCR for butyrate-producing bacteria

The final step from butyryl-CoA to butyrate is either catalyzed by butyrate kinase or butyryl-CoA kinase. Typically, these two genes are used as biomarkers for the identification/detection of butyrate-producing communities^52^. Targeting the whole pathway for functional predictions is a robust way to circumvent difficulties associated with the analysis based on specific genes only^53, 54^. The levels of *buk* and *butyryl-CoA* gene expression were normalized by total bacterial DNA and compared with high fat (HF) diabetic controls at the end of the study. Primer sequences are given in Supplementary Table S2.

### Real-time-PCR analysis

After the mice were sacrificed, total RNA was extracted from frozen tissues using Trizol reagent (Invitrogen), according to the manufacturer’s instructions. Fast-Start SYBR Green PCR reagents (Roche) were used to determine mRNA levels. Primer sequences are given in Supplementary Table S2. β-actin was used as housekeeping control. Calculations were made based on the comparative cycle threshold method (2−DDCt).

### Biochemical analyses

After the mice were sacrificed, HbA1C, ALT, AST, ALP, TG, High density lipoprotein cholesterol (HDL-C), LDL-C, Total cholesterol (CHOL) and fructosamine were measured by biochemical analyzer (Mindray BS-480, Shenzhen, China).

### ELISA

Serum G6Pase, insulin, GLP-1, TNF-α were measured by ELISA (Wenle, Shanghai, China) according to the manufacturer’s instructions. TNF-α, IL-6 and MCP-1 in adipose were measured using an ELISA kit (R&D, Minneapolis, USA) according to the standard procedure. After the mice were sacrificed, for liver, pancreas, adipose, colon tissue samples (G6Pase, GLP-1, TNF-α, IL-6 and MCP-1 detection), the tissue was homogenized with PBS, then the homogenate was centrifuged at 4 °C for 10 min at 4000 g, supernatant was used for ELISA analysis.

### Western blot analysis

Liver and adipose samples were obtained after fasting for 6 h, lysed with RIPA buffer (containing protease inhibitors, Beyotime, Shanghai), then grinded with high-throughput tissue burnisher (SCIENTZ-48, Ningbo). The homogenates were centrifuged at 4 °C for 15 min at 8,000 × g and supernatants were used for western blot. Protein concentration was quantified using a BCA protein assay Kit (Beyotime, Shanghai) and then equal amounts (100 μg) of total proteins were loaded on a polyacrylamide SDS–PAGE gel. Proteins were transferred to a PVDF membrane, which were then blocked with blocking buffer for 1 h at room temperature followed by incubation overnight at 4 °C with appropriate antibodies. β-actin was purchased from BOSTER (Wuhan, China). pIRS-1-Ser307 was purchased from ABclonal (Boston, MA, USA). pAKT-Ser473, AKT, PPARγ, PGC1α, CPT1α, Nrf2, UCP1 and pP38 antibodies were purchased from Cell Signaling Technology (Beverly, MA, USA). Incubation with fluorescently labeled horseradish peroxidase (HRP)-conjugated secondary antibodies (1:5,000) was performed for 2 h at room temperature. Immunoreactivity was analyzed using Western Lightening Plus-ECL (Pierce, Rockford, IL, USA) according to the manufacturer’s instructions.

### SCFA analysis

Acetate, propionate and butyrate in stool samples were analyzed by gas chromatography coupled mass spectrometry (GC-MS). At the end of the study, stool samples were collected and immediately stored at −80 °C. Stool samples (50 mg) were first homogenized in 500 μl of saturated NaCl solution, then acidified with 40 μl of 10% sulfuric acid. After that, 1 ml diethyl ether was added to the samples to extract SCFAs, then samples were centrifuged at 14,000 *g*, 4 °C, 15 min, and the supernatant was used for GC-MS^55^.

### Statistics

All data are presented as mean ± SEM. One-way analysis of variance (ANOVA) was performed to determine the significance among three or more groups followed by the indicated post hoc test. Independent *t*-test was used for two independent groups. *p* < 0.05 was considered statistically significant. All data were analyzed using GraphPad Prism 5 software (San Diego, CA, USA).

## Electronic supplementary material

Supplementary Information
