# Supplementary material for: Anti-diabetic Effects of Clostridium butyricum CGMCC0313.1 through Promoting the Growth of Gut Butyrate-producing Bacteria in Type 2 Diabetic Mice
Source: Sci Rep. 2017 Aug 1;7:7046. doi: 10.1038/s41598-017-07335-0 (PMC5539151; doi:10.1038/s41598-017-07335-0)
Supplement: Supplementary file 1 — Supplementary Information [file 41598_2017_7335_MOESM1_ESM.pdf]

Supplementary Information: Anti-diabetic effects of *Clostridium butyricum* CGMCC0313.1 through promoting the growth of gut butyrate-producing bacteria in Type 2 Diabetic Mice

Lingling Jia[1,2], Dongyao Li[1], Ninghan Feng[2,3], Muhammad Shamoon[1], Zhenghua Sun[1], Lei Ding[4], Hao Zhang[1], Wei Chen[1], Jia Sun[1*] & Yong Q Chen[1,2,5*]

[1]State Key Laboratory of Food Science and Technology, School of Food Science and Technology, Jiangnan University, Wuxi 214122, P. R. China.

[2]Wuxi School of Medicine, Jiangnan University, Wuxi 214122, Jiangsu, P. R. China.

[3]Wuxi No. 2 Hospital, Jiangsu, P. R. China.

[4]Department of Biology and Chemistry, University Bremen. Leobener Str., NW 2, 28359 Bremen, Germany.

[5]Department of Cancer Biology, Wake Forest School of Medicine, Winston-Salem, NC 27157, USA

*Corresponding authors: State Key Laboratory of Food Science and Technology, School of Food Science and Technology, E-mail: jiasun@jiangnan.edu.cn or Wuxi School of Medicine, Jiangnan University, Wuxi 214122, P. R. China. E-mail: yqchen@jiangnan.edu.cn. Tel/Fax: +86 (0)510 85197370.

Supplementary Table S1-Rodent Diet with 60% kcal fat (D12492)

Product		gm%	kcal%
Protein		26.2	20
Carbohydrate		26.3	20
Fat		34.9	60
	Total		100
	kcal/gm	5.24	
Ingredient		gm	kcal
Casein, 80 Mesh		200	800
L-Cystine		3	12
Corn Starch		0	0
Maltodextrin 10		125	500
Sucrose		68.8	275.2
Cellulose, BW200		50	0
Soybean Oil		25	225
Lard*		245	2205
Mineral Mix, S10026		10	0
DiCalcium Phosphate		13	0
Calcium Carbonate		5.5	0
Potassium Citrate1 H2O		16.5	0
Vitamin Mix, V10001		10	40
Choline Bitartrate		2	0

FD&C Blue Dye #1	0.05	0
Total	773.85	4057

Supplementary Table S2-Primer sequences used in this study for gene expression and microbial abundance

Gene name	Gene symbol	Sequence
butyrate kinase gene	*buk*	Buk-5F1 CCATGCATTAAATCAAAAAGC
		Buk-5F2 CCATGCGTTAAACCAAAAAGC
		Buk-6R1 AGTACCTCCACCCATGTG
		Buk-6R2 AATACCTCCGCCCATATG
		Buk-6R3 AATACCGCCRCCCATATG
Total bacteria	Total	Forward5'- GCAGGCCTAACACATGCAAGTC
		Reverse 5'-CTGCTGCCTCCCGTAGGAGT
butyryl-coenzyme A gene	*but-CoA*	Forward5'-GCIGAICATTTCACITGGAAYWSITGGCAYATG
		Reverse 5'-CCTGCCTTTGCAATRTCIACRAANGC
Clostridium butyrate CGMCC0313.1	CB0313.*1*	Forward5'-CCTCCTTTCTATGGAGAAATCTAGCA
		Reverse 5'-TGTAGCTTGACCTTTTTAAGTTTTGA
Free fatty acid receptor type 1	*Ffar1*	Forward 5'-CCTTCGCTCTCTATGTATCTGCC-3'
		Reverse 5'-CGCAGTTTAGCGTGGGACA-3'

Free fatty acid	*Ffar2*	Forward 5’-ATCCTCCTGCTTAATCTGACCC-3’
receptor type 2		Reverse 5’-CGCACACGATCTTTGGTAGGT-3’
Free fatty acid	*Ffar3*	Forward 5’-CTTCTTTCTTGGCAATTACTGGC-3’
receptor type 3		Reverse 5’-CCGAAATGGTCAGGTTTAGCAA-3’
Glp-1 receptor	*Glp1R*	Forward 5’-ACGGTGTCCCTCTCAGAGAC-3’
		Reverse 5’-ATCAAAGGTCCGGTTGCAGAA-3’
Bacteriodetes	Bac	Forward 5’-GAAGGTCCCCCACATTG-3’
		Reverse 5’-CAATCGGAGTTCTTCGTG-3’
Firmicutes	Firm	Forward
		5’-GGAGYATGTGGTTTAATTCGAAGCA-3’
		Reverse 5’-AGCTGACGACAACCATGCAC-3’
Lactobacilli	Lac	Forward 5’-AGCAGTAGGGAATCTTCCA-3’
		Reverse 5’-CACCGCTACACATGGAG-3’
Bifidobacteria	Bifid	Forward 5’-GCGTGCTTAACACATGCAAGTC-3’
		Reverse 5’-CACCCGTTTCCAGGAGCTATT-3’
Universal	Univ	Forward 5’-TCCTACGGGAGGCAGCAGT-3’
		Reverse 5’-GACTACCAGGGTATCTAATCCTGTT-3’
Clostridial	XIVa	Forward5’- CGGTACCTGACTAAGAAGC
cluster XIVa		Reverse 5’-AGTTTYATTCTTGCGAACG
Clostridial	IV	Forward5’-TTA CTG GGT GTA AAG GG
cluster IV		Reverse 5’-TAG AGT GCT CTT GCG TA
β-actin		Forward5’-CCCAGGCATTGCTGACAGG

	Reverse 5’-TGGAAGGTGGACAGTGAGGC
Cramp	Forward5’-CTTCAAGGAACAGGGGGTGG
	Reverse 5’-CTTGAACCGAAAGGGCTGTG
G6pase	Forward5’-ATGGAGGAAGGAATGAACAT
	Reverse 5’-TGGGAGTCTTGGTAATTCAC
Pck1	Forward5’-GAGGAGCTGTTTGGGATCTC
	Reverse 5’-TTGACCTGGTCCTCCAGATA
Glut1	Forward5’- GCAGAGGCTTGCTTGTAGAG
	Reverse 5’- AACGGACGCGCTGTAACTAT
Glut4	Forward5’- CTCATTCTTGGACGGTTCCT
	Reverse 5’- GCGATTTCTCCCACATACAT
Ucp-1	Forward5’- AAACAGAAGGATTGCCGAAA
	Reverse 5’- AATCCTGAGTGAGGCAAAGC
Acc1	Forward5’- TGTCCGCACTGACTGTAACC
	Reverse 5’- ATGCTCCGCACAGATTCTTC
Gk	Forward5’- GCAGAAGGGAACAACATCGT
	Reverse 5’- ATTGCCACCACATCCATCTC

Supplementary Table S3-The alteration of feces microbiota composition messured by metagenome.

Values presented here are mean ± SD (n=5-6). #, ##, ### different in comparison with HFD by One-way ANOVA followed by the indicated post hoc test. *, **,***,

different in comparison with HFD by *t* tests.

Taxa (%)	HFD	CB	NaB	ND
Phylum				
Bacteroidetes	5.68 ±1.38	16.42 ±4.19*	6.97 ±4.57	48.68 ±6.16$^{\#\#\#}$
Firmicutes	88.42 ±8.16	72.78 ±13.39$^{\#}$	86.82 ±5.83	47.90 ±8.07$^{\#\#\#}$
Class				
Bacteroidia	5.68 ±1.36	16.48 ±13.46*	6.96 ±4.57	48.63 ±8.89$^{\#\#\#}$
Bacilli	5.96 ±4.71	8.72 ±7.72	11.57 ±4.11	17.68 ±6.90
Clostridia	32.97 ±3.73	56.53±16.69*	41.30 ±14.64	25.50 ±7.57*
Erysipelotrichi	48.67 ±5.03	6.67 ±4.27$^{\#\#\#}$	32.87 ±2.67	4.35 ±10.21$^{\#\#\#}$
Epsilonproteobacteria	0.28 ±0.29	3.66 ±3.38	1.46 ±2.55	0.81 ±0.82
Order				
Coriobacteriales	2.02 ±3.58	0.78 ±2.51	1.46 ±0.70	0.84 ±1.22$^{\#\#\#}$
Bacteroidales	5.68 ±1.34	16.41 ±4.09	6.96 ±4.22	48.63 ±5.32$^{\#\#\#}$
Lactobacillales	5.44 ±4.74	8.29 ±7.76	11.43 ±4.18	12.79 ±6.88
Clostridiales	32.95 ±3.33	56.53 ±13.44*	41.29 ±12.02	25.45 ±7.57
Erysipelotrichales	48.67 ±5.03	6.76 ±4.27$^{\#\#\#}$	32.87 ±2.67*	4.35 ±10.21$^{\#\#\#}$
Family				
Coriobacteriaceae	3.15 ±2.96	0.88 ±0.69	1.37 ±0.78	1.12 ±1.22
Bacteroidaceae	0.53 ±0.39	1.68 ±0.99$^{\#}$	0.75 ±0.49	0.58 ±0.28
Porphyromonadace	7.48 ±4.22	9.42 ±8.08	7.43 ±3.69	14.66 ±6.88

ae				
Lachnospiraceae	6.97 ±1.22	12.57 ±4.18#	8.59 ±4.52	6.32 ±2.40
Ruminococcaceae	8.72 ±2.23	16.58 ±36.30*	15.03 ±6.24*	3.46 ±1.28**
Rikenellaceae	1.37 ±0.31	3.64 ±3.10	2.55 ±3.14	14.28 ±6.57###
Deferribacteraceae	0.15 ±0.12	1.18 ±1.49	0.82 ±1.34	0.14 ±0.11
Lactobacillaceae	7.48 ±4.22	9.42 ±8.08	7.43 ±3.69	14.66 ±6.88
Helicobacteraceae	0.37 ±0.23	3.12 ±3.36	2.03 ±2.44	0.86 ±0.82
Erysipelotrichaceae	42.03 ±7.96	8.15 ±5.07###	31.60 ±15.69	5.46 ±4.49###
Clostridiaceae	0.30 ±0.00	3.98 ±4.26#	0.23 ±0.19	0.80 ±0.55
Genus				
Akkermansia	0.66 ±0.46	1.54 ±1.93	0.82 ±0.66	0.24 ±0.48
Ruminococcus	0.48 ±0.14	1.10 ±0.38#	0.88 ±0.34#	0.30 ±0.12
Bacteroides	0.62 ±0.35	1.68 ±0.99#	0.75 ±0.49	0.58 ±0.29
S24-7	2.45 ±1.00	10.05 ±11.09	3.17 ±1.60	38 ±12.53###
Clostridium	0.00 ±0.00	4.43 ±4.55#	0.00 ±0.00	0.50 ±0.48
Streptococcus	1.27 ±0.27	2.22 ±1.09	2.47 ±0.54##	0.12 ±0.11#
Mucispirillum	0.25 ±0.12	1.49 ±1.49	0.70 ±1.34	0.10 ±0.11
Oscillospira	7.05 ±1.85	12.70 ±5.38*	11.83 ±5.17*	1.80 ±0.53***
f-Rikenellaceae, g-	1.32 ±0.26	3.48 ±3.09	2.47 ±3.07	13.90 ±6.49###
Allobaculum	48.51 ±8.02	6.44 ±5.21###	32.81 ±15.7	4.06 ±4.28###
Lactobacillus	7.42 ±4.16	9.37 ±8.09	7.38 ±3.68	12.49 ±6.85

Butyricimonas	0.10 ±0.09	0.34 ±0.28	0.40 ±0.21*	0.22 ±0.13
Odoribacter	0.51 ±0.29	1.83 ±2.11	0.40 ±0.60	1.83 ±0.95*

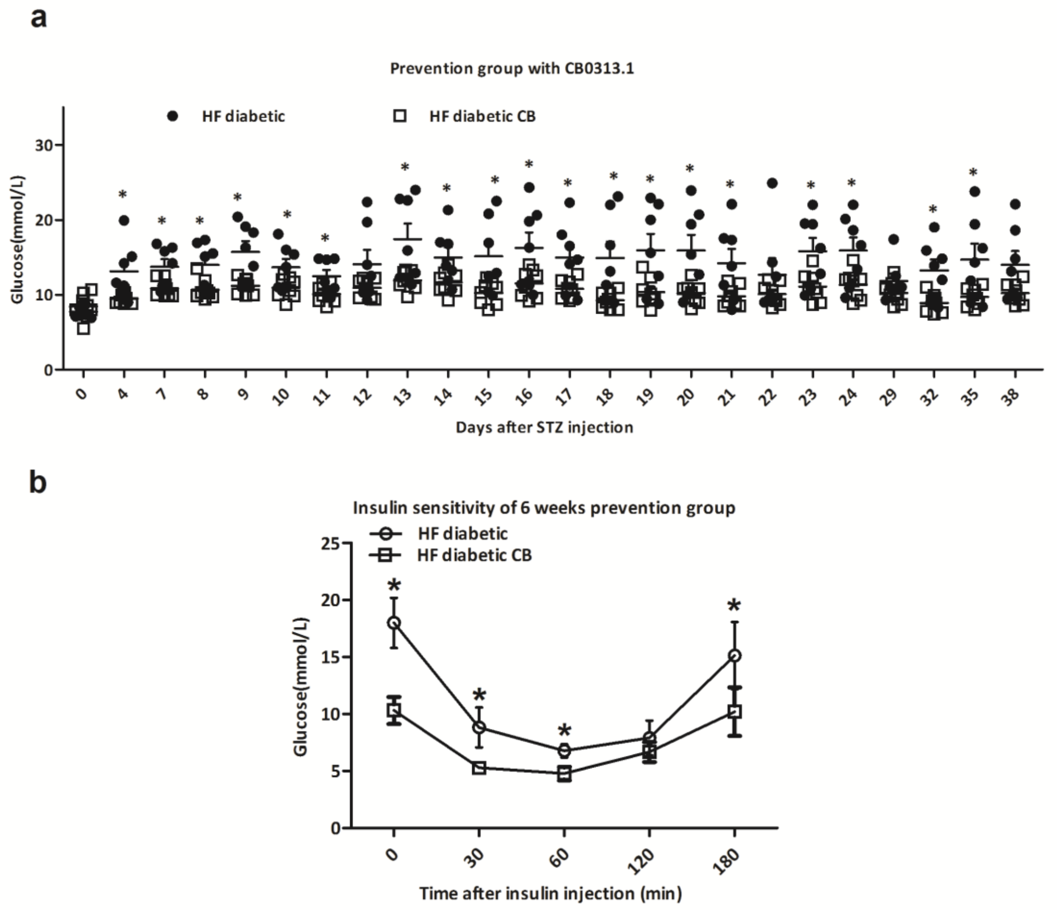

Figure S1. CB0313.1 ameliorates glucose homeostasis in HF diabetic mice-the detailed results in 2014. (**a**) Glucose was tested after 6h fasting; (**b**) Insulin tolerance test; *HF diabetic* high fat diet and streptozotocin, *HF diabetic CB* high fat diet and streptozotocin plus CB0313.1. Data are mean ± SEM, (n = 3-8 mice per group). $^{\#}$, $^{\#\#}$, $^{\#\#\#}$ p<0.05, p<0.01, p<0.001 *vs* HF diabetic control by One-way ANOVA followed by the indicated post hoc test. *, **, *** p<0.05, p<0.01, p<0.001 *vs* HF diabetic control by *t*-test. *HF diabetic:* high fat diet and streptozotocin, *HF diabetic CB:* high fat diet and streptozotocin plus CB0313.1

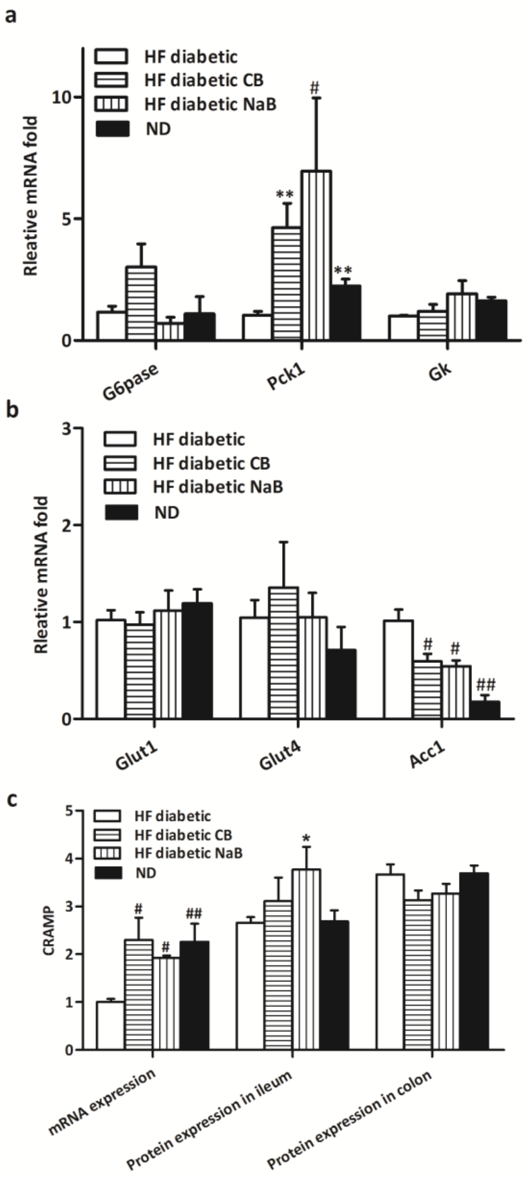

Figure S2. CB0313.1 activates intestinal gluconeogenesis and increase cathelicidin related antimicrobial peptide (CRAMP) secretion in ileum in HF diabetic mice. (**a**) *G6pase, Pck1 and Gk* mRNA in colon; (**b**) *Glut1, Glut4 and Acc1* mRNA in colon; (**c**) CRAMP expression at mRNA and protein. *HF diabetic* high fat diet and streptozotocin, *HF diabetic CB* high fat diet and streptozotocin plus CB0313.1, *HF diabetic NaB HF diabetic CB* high fat diet and streptozotocin plus NaB, *ND* Normal diet. Data are mean ± SEM, (n = 3-12 mice per group). $^{\#}$, $^{\#\#}$, $^{\#\#\#}$ p<0.05, p<0.01, p<0.001 *vs* HF diabetic control by One-way ANOVA followed by the indicated post hoc test. *, **, *** p<0.05, p<0.01, p<0.001 *vs* HF diabetic control by *t*-test. *HF diabetic:* high fat diet and streptozotocin, *HF diabetic CB:* high fat diet and streptozotocin plus CB0313.1, *HF diabetic* NaB*:* high fat diet and streptozotocin plus NaB, *ND:* Normal diet.

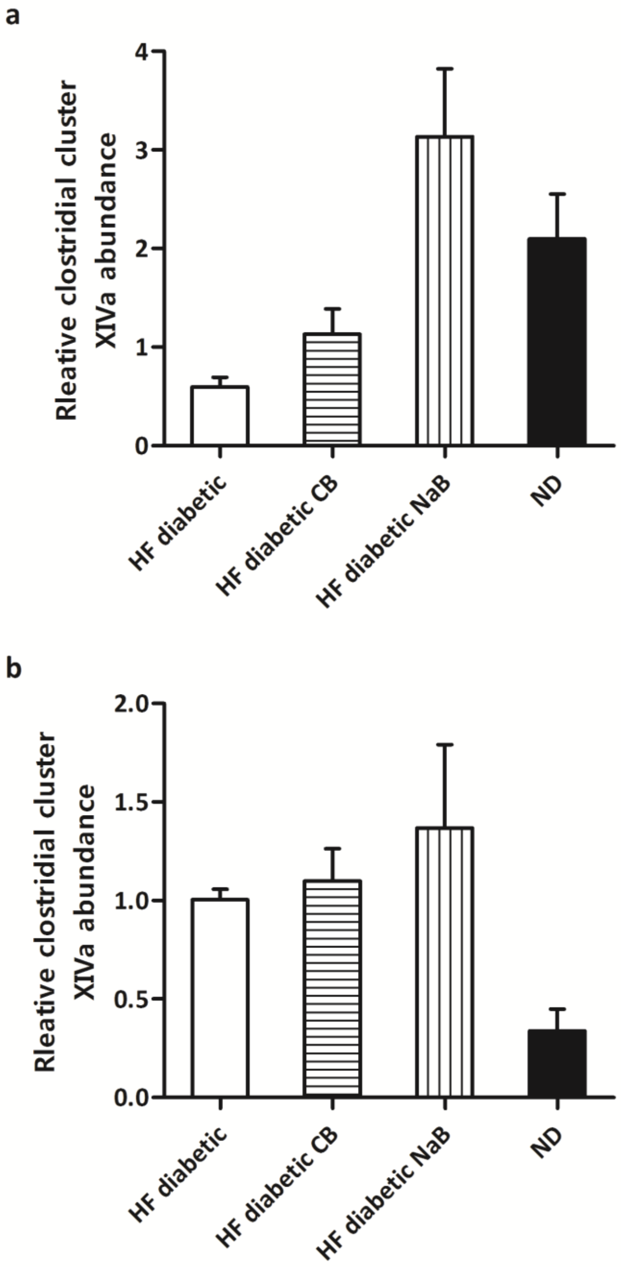

Figure S3. (**a**) Relative clostridial cluster IV abundance; (**b**) Relative clostridial cluster XIVa abundance. *HF diabetic* high fat diet and streptozotocin, *HF diabetic CB* high fat diet and streptozotocin plus CB0313.1, *HF diabetic NaB HF diabetic CB* high fat diet and streptozotocin plus NaB, *ND* Normal diet. Data are mean ± SEM, (n = 3-5 mice per group). $^{\#}$, $^{\#\#}$, $^{\#\#\#}$ *p*<0.05, *p*<0.01, *p*<0.001 *vs* HF diabetic control by One-way ANOVA followed by the indicated post hoc test. *, **, *** *p*<0.05, *p*<0.01, *p*<0.001 *vs* HF diabetic control by *t*-test. *HF diabetic:* high fat diet and streptozotocin, *HF diabetic CB:* high fat diet and streptozotocin plus CB0313.1, *HF diabetic* NaB*:* high fat diet and streptozotocin plus NaB, *ND:* Normal diet.

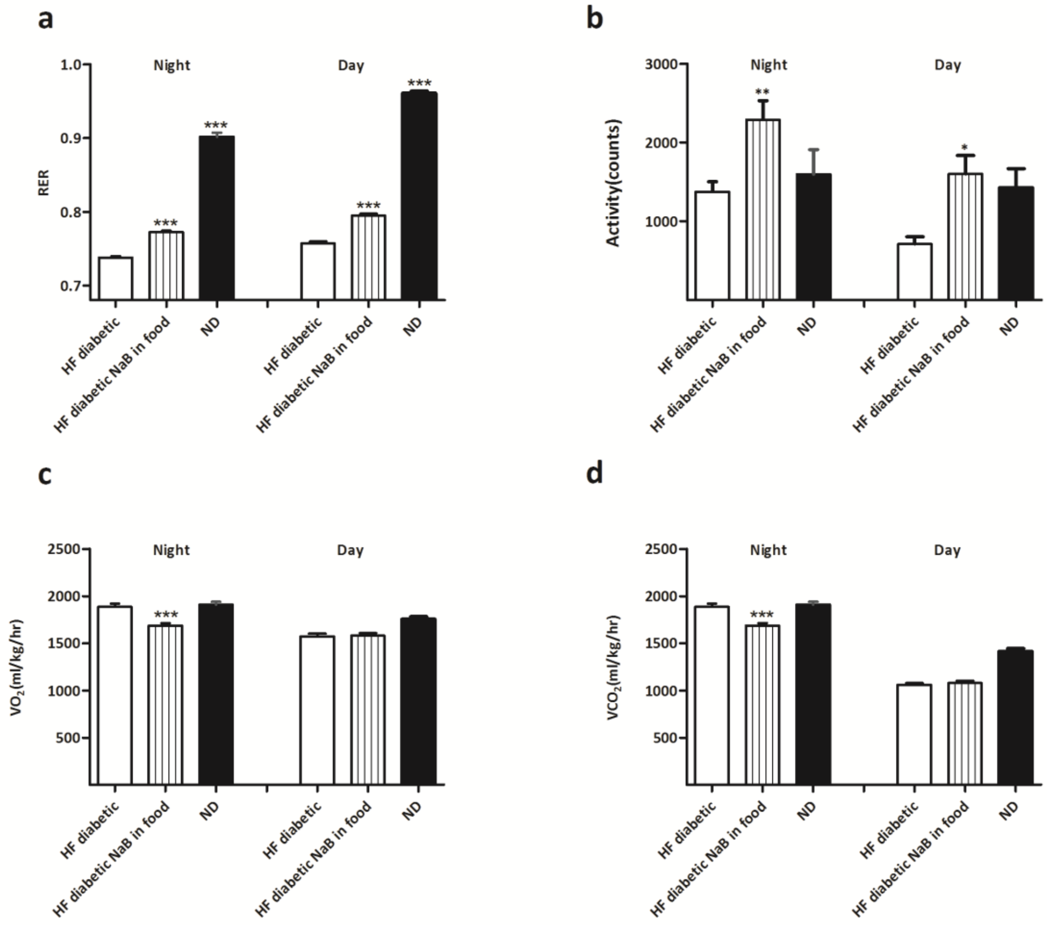

Figure S4. We also incorporated sodium butyrate into HF diet at 5% w/w as a supplemental experiment. The Sodium butyrate containing diet was pelleted and stored in a -20 ℃ freezer until usage. Energy expenditure was examined using a metabolic chamber at 17 weeks of age. (**a**) Substrate utilization is expressed by respiratory exchange ratio (RER), the ratio of O_2 consumption *vs* CO_2 exhalation volume; (**b**) Spontaneous physical activity; (**c**) VO_2; (**d**) VCO_2. Data are mean ± SEM, (n = 4 mice per group). $^{\#}$, $^{\#\#}$, $^{\#\#\#}$ $p<0.05$, $p<0.01$, $p<0.001$ *vs* HF diabetic control by One-way ANOVA followed by the indicated post hoc test. *, **, *** $p<0.05$, $p<0.01$, $p<0.001$ *vs* HF diabetic control by *t*-test. *HF diabetic:* high fat diet and streptozotocin, *HF diabetic* NaB*:* high fat diet and streptozotocin plus NaB, *ND:* Normal diet.

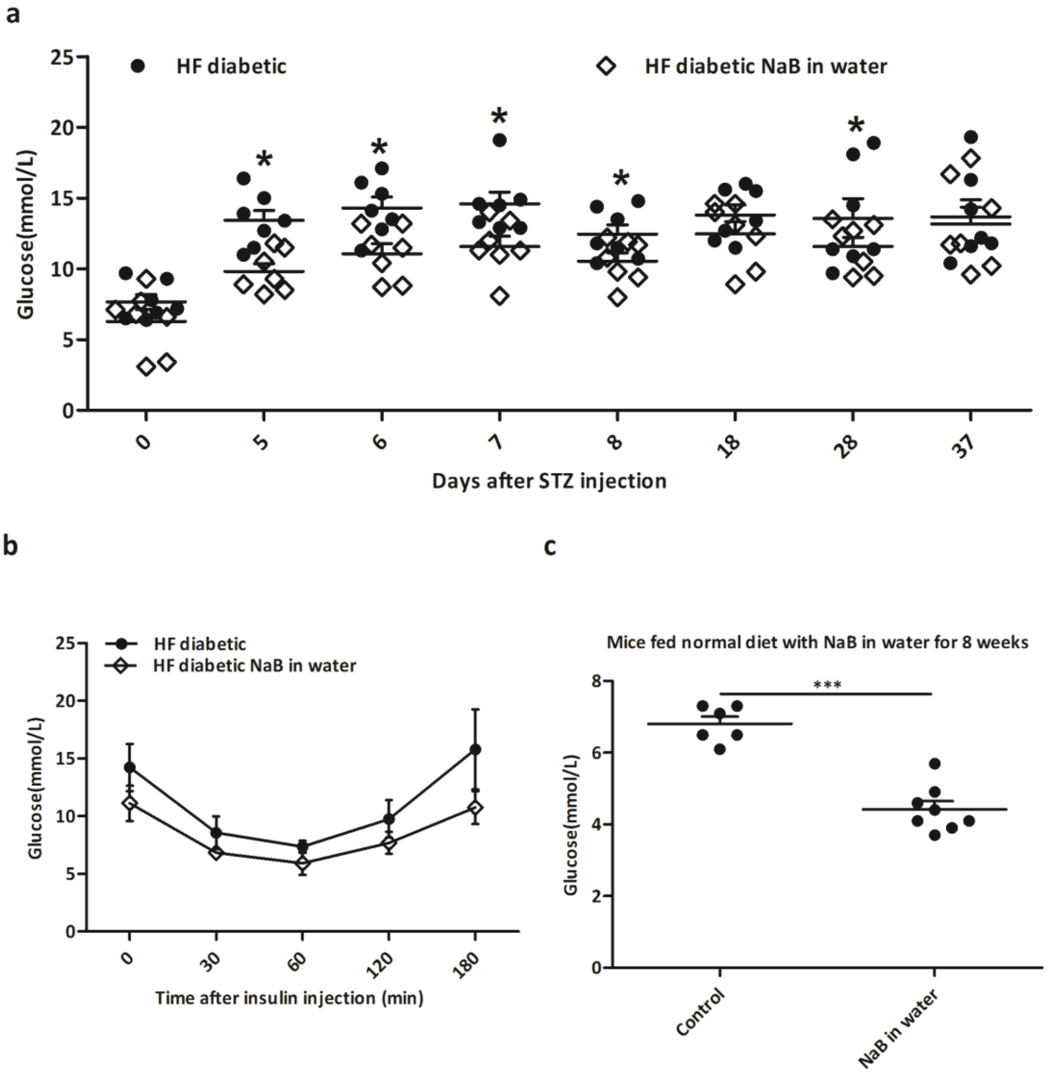

Figure S5. NaB in water improves fasting hyperglycemia and insulin resistance-the results in 2014. We also incorporated sodium butyrate into water at 1% as a supplemental experiment. (**a**) Glucose was tested after 6h fasting. (**b**) Insulin tolerance test. (**c**) Glucose was tested after 6h fasting. Data are mean ± SEM, (n = 3-8 mice per group). $^{\#}$, $^{\#\#}$, $^{\#\#\#}$ $p<0.05$, $p<0.01$, $p<0.001$ *vs* HF diabetic control by One-way ANOVA followed by the indicated post hoc test. *, **, *** $p<0.05$, $p<0.01$, $p<0.001$ *vs* HF diabetic control by *t*-test. *HF diabetic:* high fat diet and streptozotocin, *HF diabetic* NaB*:* high fat diet and streptozotocin plus NaB, *ND:* Normal diet.
